# The hierarchical scaffolding model of executive functioning in university students: a self-reported explanatory approach

**DOI:** 10.3389/fpsyg.2026.1906868

**Published:** 2026-07-29

**Authors:** Diego D. Díaz-Guerra, Marena de la C. Hernández-Lugo, Carlos Ramos-Galarza, Yunier Broche-Pérez, Hugo Arias-Flores, Katiuska Viviana Carranza-Reinado, Evelyn Fernández-Castillo

**Affiliations:** 1 Departamento de Psicología, Facultad de Ciencias Sociales, Universidad Central “Marta Abreu” de Las Villas, Santa Clara, Villa Clara, Cuba; 2 Departamento de Psicología-Sociología, Facultad de Ciencias Sociales, Universidad de Camagüey “Ignacio Agramonte Loynaz”, Camagüey, Cuba; 3 Centro de Bienestar Universitario, Departamento de Psicología, Facultad de Ciencias Sociales, Universidad Central “Marta Abreu” de Las Villas, Santa Clara, Villa Clara, Cuba; 4 Carrera de Psicología Clínica, Facultad de Salud y Bienestar, Pontificia Universidad Católica del Ecuador, Quito, Ecuador; 5 Department of Behavior Analysis, Prisma Behavioral Center, West Palm Beach, FL, United States; 6 Centro de Investigación de Ciencias Humanas y de la Educación (CICHE), Universidad Tecnológica Indoamérica, Quito, Ecuador; 7 Facultad de Ciencias de la Salud, Universidad Laica Eloy Alfaro de Manabí, Manta, Manabí, Ecuador

**Keywords:** decision trees, executive functions, explanatory model, structural equation modeling, UEF-1, university students

## Abstract

**Introduction:**

Executive functions are cognitive processes that are fundamental to academic success among university students. However, most explanatory models have been developed using laboratory-based tasks, which limits their ecological validity. Self-report measures offer a complementary approach by capturing perceived executive functioning in real academic contexts.

**Objective:**

To construct and validate an explanatory model of executive functioning in Cuban university students.

**Methods:**

A cross-sectional sample of 1,233 students was analyzed (mean age = 20.08 years, SD = ±1.02; 59.3% women). The Cuban adaptation of the UEF-1 was administered. Analyses combined decision trees using the CHAID method to identify predictors and structural equation modeling (SEM) to confirm the relationships among variables and construct a unified model.

**Results:**

The final model showed satisfactory fit (CFI = 0.915, RMSEA = 0.048, SRMR = 0.045). Executive functions were organized into four hierarchical levels: (1) Metacognitive Evaluation and Supervision, comprising Conscious Monitoring of Responsibilities and Behavioral Verification for Learning; (2) Emotional and Behavioral Regulation, comprising Conscious Regulation of Emotions and Conscious Regulation of Behavior; (3) the Operational Attentional System; and (4) Goal-Directed Action and Task Resolution, comprising Decision-Making and Management of Elements to Solve Tasks.

**Conclusion:**

The proposed model constitutes the first unified explanatory framework of executive functioning in Cuban university students, specifically as assessed through self-report measures. Its four-level hierarchical structure provides an empirically grounded guide for future research and interventions, capturing the subjective dimension of executive performance as a complementary and ecologically relevant level of analysis closely tied to academic adaptation and self-regulation.

## 1 Introduction

Executive functions comprise a set of higher-order cognitive processes that enable individuals to plan, regulate, and monitor goal-directed behavior, particularly in novel or complex contexts that require conscious and deliberate control of action (Diamond, 2013; Friedman and Miyake, 2017). In the university setting, these capacities are essential for academic success, as students must autonomously manage their responsibilities, sustain attention for prolonged periods, regulate emotional states in response to evaluative pressure, and make strategic decisions about allocating their cognitive and temporal resources (Wong Carriera et al., 2025). Despite growing interest in understanding the role of executive functions in self-regulated learning (Dörrenbächer-Ulrich and Bregulla, 2024), most studies have focused on child or clinical populations (Folker et al., 2025; Tervo-Clemmens et al., 2023), whereas research on neurotypical young adults, particularly in Latin American contexts, remains comparatively limited.

Traditionally, explanatory models of executive functions have fluctuated between two main approaches. On the one hand, factorial models, inspired by the seminal work of Miyake et al. (2000), propose a unity-and-diversity structure in which relatively independent components, such as working memory updating, inhibition, and attentional shifting, correlate with one another while retaining their own specific characteristics (Friedman and Miyake, 2017). On the other hand, hierarchical models propose a pyramidal organization in which higher-order metacognitive processes supervise and coordinate more basic executive functions (Stuss, 2011; Zelazo and Carlson, 2012). However, a recurrent limitation of these models is that they have been developed predominantly from laboratory-based experimental studies using isolated tasks. This limits their ecological validity for explaining executive functioning in real academic contexts (Dawson, 2014; Dawson and Guare, 2018), where multiple demands converge simultaneously and students must dynamically integrate cognitive, emotional, and behavioral competencies (Damayanti, 2026).

In response to this limitation, the development of self-report instruments capable of capturing executive functioning in everyday situations, including the university context, has gained increasing relevance in recent years. The University Executive Functions Scale (UEF-1), designed and validated by Ramos-Galarza et al. (2023) in Chilean and Ecuadorian populations, represents an important advance in this direction by assessing seven executive functions specifically theorized for university students. Recently, Díaz-Guerra et al. (2025a) validated this instrument in a Cuban university population, opening the possibility of examining how these executive functions are articulated within a specific sociocultural context characterized by particular educational conditions that may modulate the expression and organization of executive control.

The use of self-report measures to assess executive functions requires careful consideration of what is being measured. According to Barkley's (2012) extended phenotype model of executive functioning, self-report instruments and performance-based tests assess different but complementary levels of the executive system. Performance-based tests primarily evaluate the Instrumental–Self-Directed level, capturing cognitive components such as inhibition, working memory, and planning in controlled, short-term contexts. In contrast, self-report measures and ecological observations are more likely to assess the Methodical–Autonomous, Tactical–Reciprocal, and Strategic–Cooperative levels of executive functioning, which unfold over days, weeks, and months, and are more closely related to real-world adaptive functioning and impairments in major life activities (Barkley, 2012). Thus, while the present study captures students’ perceptions and self-assessments of their executive abilities rather than objective performance on neuropsychological tasks, these perceptions are not merely subjective artifacts; they reflect higher-order, ecologically valid levels of the extended executive phenotype that are essential for understanding self-regulation in naturalistic academic contexts. Consequently, the model proposed here should be understood as representing the perceived organization of executive functions at these higher phenotypic levels, complementing rather than competing with performance-based approaches.

A recent and relevant contribution to the development of explanatory models in this field is the work of Díaz-Guerra et al. (2025b), who used the same UEF-1 scale in Cuban university students and applied a combined methodology involving decision trees and structural equation modeling to explore predictive interrelations among executive functions. This study identified the Supervisory Attentional System as the main predictor of Conscious Monitoring of Responsibilities, whereas Conscious Regulation of Emotions emerged as the strongest predictor of Conscious Regulation of Behavior. However, this initial approach to an explanatory model had two substantial limitations. First, it focused exclusively on two executive functions—Conscious Monitoring of Responsibilities and Conscious Regulation of Behavior—without integrating all seven functions assessed by the instrument into a unified framework. Second, although it identified relevant predictive relationships, it did not propose a hierarchical architecture specifying levels of processing or the direction of information flow among all executive functions operating concurrently when students face academic demands.

Despite these initial advances, a substantial gap remains in the literature. To date, there is no comprehensive explanatory model that describes how ecologically assessed executive functions, as perceived and reported by the students themselves, are interrelated when they face the academic demands of everyday university life, a level of analysis that is essential for understanding executive control in real-world contexts. In Cuba, most studies have been limited to correlational or comparative analyses, without exploring directions of influence, mediating effects, or the functional architecture underlying executive performance in natural educational contexts (Blanco Martí et al., 2026; Jiménez-Puig et al., 2019).

This theoretical gap has important practical implications, as it hinders the design of interventions to strengthen executive functioning among university populations. Without a clear map of which functions constitute critical nodes within the system, and how changes in one component may transfer to others, intervention planning remains limited. Therefore, the present study aims to construct an explanatory model that integrates the seven executive functions assessed by the UEF-1 in Cuban university students, as perceived and self-reported by students in their academic contexts, a complementary and ecologically grounded level of analysis that reflects how executive control is experienced and organized in real-world educational settings. Given that the previous literature does not offer a single framework that articulates these functions into a cohesive system for university populations, an inductive model-building strategy was adopted, in which the final architecture is not imposed *a priori* from theory but is derived from the patterns observed in the data and subsequently interpreted in light of existing theoretical frameworks on executive control.

## 2 Methods

### 2.1 Study design and participants

The study was conducted using a cross-sectional design with a quantitative correlational approach. The sample consisted of 1,233 Cuban university students. The mean age of the sample was 20.08 years (range = 17–25, SD = ±1.02). Regarding gender, 40.7% of the participants were men and 59.3% were women. None of the participants reported a history of psychological disorders, drug use, or any health condition that could affect their executive functioning.

### 2.2 Instruments

#### 2.2.1 Demographic information

Demographic variables included age and gender.

#### 2.2.2 University Executive Functions Scale (UEF-1)

This scale was designed and validated for Spanish-speaking participants by Ramos-Galarza et al. (2023) in a sample of 1,373 Chilean and Ecuadorian students. It was later validated in the Cuban population by Díaz-Guerra et al. (2025a) in a sample of 1,092 university students. The scale consists of 31 items and assesses seven executive functions (*α* = 0.91, *ω* = 0.92). Responses are provided on a 5-point Likert scale ranging from 1 = totally disagree to 5 = totally agree. The subscales assessed by the instrument are:

*F1 (α = 0.82, ω = 0.84): Conscious Monitoring of Responsibilities*, for example, Item 2, “I can complete a university task even when it is very long,” and Item 15, “I can do my work without someone supervising me.”

*F2 (α = 0.85, ω = 0.86): Supervisory Attentional System*, for example, Item 14, “I am able to maintain attention on an activity,” and Item 22, “It is easy for me to concentrate on my university activities.”

*F3 (α = 0.65, ω = 0.69): Conscious Regulation of Behavior*, for example, Item 3, “I always act by thinking and reflecting on the consequences of my actions,” and Item 19, “I can anticipate the consequences of my actions.”

*F4 (α = 0.81, ω = 0.82): Behavioral Verification for Learning*, for example, Item 20, “I check that my university assignments are well done and error-free before submitting them to the professor,” and Item 24, “I remember to bring home university tasks, materials, or papers.”

*F5 (α = 0.72, ω = 0.73): Decision-Making*, for example, Item 5, “I have the ability to make decisions independently,” and Item 21, “I can make decisions without difficulty, even when facing the most complicated things.”

*F6 (α = 0.75, ω = 0.75): Conscious Regulation of Emotion*, for example, Item 25, “I remain calm easily,” and Item 29, “I have a stable mood.”

*F7 (α = 0.76, ω = 0.76): Management of Elements to Solve Tasks*, for example, Item 6, “I keep my things in the right place and in order,” and Item 7, “It is easy for me to quickly locate my materials when I look for them in my room or at my desk.”

### 2.3 Data analysis procedures

The survey was administered via Google Forms from January to December 2024 and was distributed through WhatsApp groups, email lists, and institutional websites. No incentives were offered to participants. Informed consent was obtained from each participant, and all procedures conducted in this study followed the ethical standards of the Declaration of Helsinki and its subsequent amendments. The Ethics Committee of the Department of Psychology of the Faculty of Social Sciences at Universidad Central “Marta Abreu” de Las Villas approved the study protocol under registration number AN17FEB192025. The collected data are openly available in the Mendeley Data Repository (doi: https://doi.org/10.17632/53d35x9m5m.1).

Data were processed using SPSS version 25.0 and AMOS version 22.0. Descriptive analyses were conducted to characterize the participants. Based on the national diagnostic assessment conducted in the previous stage, seven independent models were constructed, one for each executive function. On the basis of the most significant interconnections, a unified explanatory model was then developed to describe how the executive process is articulated.

To identify predictive relationships among variables in an exploratory phase, a decision tree analysis was performed. Several tree-based algorithms were considered, including Classification and Regression Trees (CART), Quick, Unbiased, Efficient Statistical Trees (QUEST), and Chi-Square Automatic Interaction Detection (CHAID). CHAID was ultimately selected for three primary reasons. First, unlike CART (which typically requires continuous or binary dependent variables and uses variance reduction or the Gini index as splitting criteria), CHAID is specifically designed to work with categorical or ordinal dependent variables (Mienye and Jere, 2024), which is appropriate for the ordinal nature of our Likert-scale data. Second, CHAID uses the chi-square test (*χ*^2^) as the splitting criterion, allowing for the detection of nonlinear interactions and non-monotonic relationships between predictor variables and the dependent variable. This feature distinguishes CHAID from algorithms such as CART and QUEST, which may be less sensitive to such complex patterns due to their reliance on variance-based or other splitting criteria. Third, CHAID can perform multiway splits, facilitating the identification of complex patterns in the data without the need to transform continuous variables into dichotomous ones, a flexibility not inherent in CART’s binary splitting approach or QUEST’s univariate splits. To evaluate the reliability of the model, the overall classification percentage and the risk estimator were considered (Mienye and Jere, 2024).

In order to minimize the risk of identifying spurious associations during the exploratory phase, only nodes with a significance level of *p* < 0.05 in the chi-square test were considered as predictors, and a minimum node size of 50 cases was required to avoid splits based on very small subgroups. Additionally, an internal 10-fold cross-validation procedure was applied to assess the stability of the trees.

In addition, confirmatory factor analyses (CFA) were conducted to validate the interconnections identified in the decision trees. Subsequently, a unified explanatory model was tested using structural equation modeling (SEM). This approach allows for the identification of direct relationships among variables, as well as indirect effects and interactions that may influence the phenomenon under study (Thakkar, 2020). Pearson correlation analyses were performed to evaluate the relationships among the executive functions comprising the instrument. Correlation coefficients were interpreted as small when *r* ≤ 0.10, moderate when *r* = 0.20, and large when *r* ≥ 0.30 (Funder and Ozer, 2019).

Ideal values for the Comparative Fit Index (CFI) were assumed to range between 0.90 and 0.95 (Hu and Bentler, 1999). In addition, because the study involved a large sample and nominal variables, values between 0.85 and 0.90 were also considered acceptable (Marsh et al., 2004). For the root mean square error of approximation (RMSEA), values below 0.10 were considered acceptable (Xia and Yang, 2019). Likewise, standardized root mean square residual (SRMR) values less than or equal to 0.08 were considered acceptable (Cho et al., 2020). In cases where SEM could not be conducted because only one predictor variable was present, simple linear regression was performed. In both cases, standardized (*β*) and unstandardized (*B*) coefficients for the interconnections are reported.

To evaluate the explanatory capacity of the final structural model, coefficients of determination (*R*^2^) were calculated for each endogenous variable. The coefficients of determination (*R*^2^) indicate the proportion of variance explained for each endogenous variable. *R*^2^ values are interpreted as: 0.01 = small, 0.09 = moderate, 0.25 = large (Cohen, 1988). Additionally, the effects among variables were decomposed into direct effects, indirect effects, and total effects.

To evaluate the measurement equivalence of the model between men and women, a multigroup invariance analysis was performed following the sequential approach recommended by Vandenberg and Lance (2000). Three levels of invariance were tested cumulatively: configural invariance, metric invariance and scalar invariance. To determine whether each level was met, the standard criteria proposed by Chen (2007) and Cheung and Rensvold (2002) were considered, where a change in CFI (ΔCFI) ≤ −0.01 between nested models indicates that invariance does not hold, while changes in RMSEA (ΔRMSEA) ≤ 0.015 complement the evaluation.

To evaluate the stability of the estimated parameters and obtain robust confidence intervals for direct and indirect effects, a bootstrapping analysis was performed with 2000 replications and a 90% confidence level, following the recommendations of Preacher and Hayes (2008) and Kline (2023). Both percentile and bias-corrected confidence intervals were used to assess the significance of structural coefficients and indirect effects. An effect was considered statistically significant if its 90% confidence interval did not include zero. All 2000 bootstrap replications were computable, with no convergence problems or singular covariance matrices, indicating the numerical stability of the model.

The combination of decision trees (CHAID) and structural equation modeling (SEM) responds to an inductive, complementary, and sequential methodological approach. Decision trees play a fundamentally exploratory and hypothesis-generating role, allowing for the identification—without prior assumptions about the form of the relationship—of which predictors are most relevant for each executive function, and detecting interactions and thresholds that might go unnoticed in a purely linear approach. SEM, for its part, fulfills a confirmatory and explanatory role by allowing the testing of relationship directionality, estimation of the magnitude of direct and indirect effects (mediations), and evaluation of the overall fit of a theoretical model that hierarchically organizes executive functions.

## 3 Results

### 3.1 Individual decision tree analyses

The decision trees revealed a systematic pattern in the predictive relationships among the executive functions (see Table 1) and allowed for the exploration of which relationships should be included in the SEM model and, crucially, how to orient the direction of the arrows in the structural model. The consistency of the pattern observed across the seven trees provided the empirical basis for the specification of the hierarchical model.

**Table 1:** Results of the decision tree analyses for each executive function.

Dependent variable	Nodes	Terminal nodes	Depth	Classification accuracy (%)	Risk (SD)	Main predictors
Conscious Monitoring of Responsibilities (F1)	10	6	3	75.0	0.25 (0.015)	F2, F4
Supervisory Attention System (F2)	14	8	3	70.3	0.29 (0.016)	F1, F3, F5
Conscious Regulation of Behavior (F3)	12	8	3	72.6	0.27 (0.015)	F6, F1
Behavior Verification for Learning (F4)	11	7	3	73.7	0.26 (0.014)	F2, F1
Decision-Making (F5)	12	7	3	70.0	0.30 (0.016)	F2, F6, F3
Conscious Regulation of Emotions (F6)	8	5	3	73.5	0.26 (0.015)	F3
Management of Elements to Solve Tasks (F7)	12	7	3	75.3	0.24 (0.014)	F3, F4

Metacognition, represented by Conscious Monitoring of Responsibilities and Behavioral Verification for Learning, consistently emerged as a fundamental level of the executive system. Conscious Monitoring of Responsibilities was identified as a predictor of Supervisory Attentional System, Conscious Regulation of Behavior, and Behavioral Verification for Learning, while Behavioral Verification for Learning predicted Conscious Monitoring of Responsibilities and Management of Elements to Solve Tasks. This suggests that the processes of conscious monitoring of responsibilities and verification of learning are positioned as a foundational level from which other components are structurally related in the proposed model. Consistently with this interpretation, the trees for Conscious Monitoring of Responsibilities and Behavioral Verification for Learning achieved the highest classification accuracies (75.0 and 73.7%, respectively), indicating that these metacognitive functions can be predicted with greater precision from other executive processes.

At the center of the architecture was situated Conscious Regulation of Behavior, which acted as the most highly connected integrating node within the system. This function was the one that appeared most frequently as a predictor across the trees: it predicted Decision-Making, Conscious Regulation of Emotions, and Management of Elements to Solve Tasks. Of particular relevance was its role as the sole predictor of Conscious Regulation of Emotions, suggesting that, within the model’s architecture, the capacity to regulate behavior is structurally positioned prior to conscious emotional regulation, rather than the reverse. The high classification accuracy of the tree for Conscious Regulation of Behavior (72.6%), together with its role as a predictor across three different executive functions, confirms its position as a central node in the organization of the executive system.

### 3.2 Individual structural equation analyses

The correlation analysis among the executive functions revealed significant associations in all cases, ranging from moderate to large in magnitude (see Table 2). Based on the relationships identified in the decision tree analyses, the predictive interrelations among the executive functions were then tested using structural equation modeling.

**Table 2:** Correlation matrix among executive functions (*n* = 1,233).

Factors	F1		F2		F3		F4		F5		F6		F7
Conscious Monitoring of Responsibilities (F1)	–												
Supervisory Attention System (F2)	0.54	**	–										
Conscious Regulation of Behavior (F3)	0.37	**	0.50	**	–								
Behavior Verification for Learning (F4)	0.45	**	0.50	**	0.37	**	–						
Decision-Making (F5)	0.40	**	0.43	**	0.41	**	0.26	**	–				
Conscious Regulation of Emotions (F6)	0.24	**	0.37	**	0.48	**	0.19	**	0.44	**	–		
Management of Elements to Solve Tasks (F7)	0.34	**	0.41	**	0.42	**	0.40	**	0.30	**	0.30	**	–

**p < 0.01.

The structural equation model for the predictors of Conscious Monitoring of Responsibilities showed an excellent level of fit based on the following values: *χ*^2^(74) = 634.667, *p* < 0.001; RMSEA = 0.07, 90% CI [0.07, 0.08], *p* < 0.001; CFI = 0.91; SRMR = 0.05. All unstandardized regression weights indicated significant relationships among the factors studied (see Table 3). Behavioral Verification for Learning had a strong positive effect on the Supervisory Attentional System (*β* = 0.670, *B* = 0.657, *p* < 0.001). In addition, the Supervisory Attentional System also had a positive effect on Conscious Monitoring of Responsibilities (*β* = 0.560, *B* = 0.427, *p* < 0.001). Finally, Behavioral Verification for Learning also positively affected Conscious Monitoring of Responsibilities (*β* = 0.310, B = 0.234, *p* < 0.001), although with a smaller effect.

**Table 3:** Unstandardized regression weights for the individual and unified models (*n* = 1,233).

Predictive relationships			*B*	S.E.	*β*	*p*
SEM for Conscious Monitoring of Responsibilities						
Supervisory Attentional System	←	Behavioral Verification for Learning	0.657	0.038	0.670	0.001
Conscious Monitoring of Responsibilities	←	Supervisory Attentional System	0.427	0.037	0.560	0.001
Conscious Monitoring of Responsibilities	←	Behavioral Verification for Learning	0.234	0.033	0.310	0.001
SEM for Supervisory Attentional System						
Conscious Regulation of Behavior	←	Decision-Making	0.646	0.056	0.767	0.001
Conscious Monitoring of Responsibilities	←	Conscious Regulation of Behavior	0.878	0.077	0.675	0.001
Supervisory Attentional System	←	Conscious Monitoring of Responsibilities	0.678	0.070	0.474	0.001
Supervisory Attentional System	←	Decision-Making	0.121	0.096	0.077	0.206
Supervisory Attentional System	←	Conscious Regulation of Behavior	0.659	0.150	0.354	0.001
SEM for Conscious Regulation of Behavior						
Conscious Regulation of Emotion	←	Conscious Monitoring of Responsibilities	0.482	0.055	0.330	0.001
Conscious Regulation of Behavior	←	Conscious Monitoring of Responsibilities	0.440	0.046	0.430	0.001
Conscious Regulation of Behavior	←	Conscious Regulation of Emotion	0.382	0.031	0.540	0.001
SEM for Behavioral Verification for Learning						
Supervisory Attentional System	←	Conscious Monitoring of Responsibilities	0.972	0.059	0.765	0.001
Behavioral Verification for Learning	←	Supervisory Attentional System	0.366	0.058	0.354	0.001
Behavioral Verification for Learning	←	Conscious Monitoring of Responsibilities	0.543	0.077	0.414	0.001
SEM for Decision-Making						
Conscious Regulation of Emotion	←	Conscious Regulation of Behavior	1.238	0.097	0.734	0.001
Supervisory Attentional System	←	Conscious Regulation of Emotion	0.501	0.036	0.542	0.001
Decision-Making	←	Supervisory Attentional System	0.213	0.029	0.308	0.001
Decision-Making	←	Conscious Regulation of Behavior	0.324	0.070	0.300	0.001
Decision-Making	←	Conscious Regulation of Emotion	0.193	0.041	0.302	0.001
SEM for Conscious Regulation of Emotion*						
Conscious Regulation of Emotion	←	Conscious Regulation of Behavior	0.512	0.026	0.489	0.001
SEM for Management of Elements to Solve Tasks						
Conscious Regulation of Behavior	←	Behavioral Verification for Learning	0.312	0.028	0.611	0.001
Management of Elements to Solve Tasks	←	Behavioral Verification for Learning	0.167	0.039	0.208	0.001
Management of Elements to Solve Tasks	←	Conscious Regulation of Behavior	0.823	0.102	0.521	0.001
SEM for the Unified Explanatory Model						
Conscious Regulation of Emotion	←	Conscious Monitoring of Responsibilities	0.464	0.048	0.358	0.001
Conscious Regulation of Behavior	←	Conscious Monitoring of Responsibilities	0.126	0.048	0.197	0.008
Conscious Regulation of Behavior	←	Conscious Regulation of Emotion	0.243	0.024	0.494	0.001
Conscious Regulation of Behavior	←	Behavioral Verification for Learning	0.222	0.045	0.380	0.001
Supervisory Attentional System	←	Behavioral Verification for Learning	0.256	0.083	0.214	0.002
Supervisory Attentional System	←	Conscious Monitoring of Responsibilities	0.581	0.084	0.445	0.001
Supervisory Attentional System	←	Conscious Regulation of Behavior	0.402	0.155	0.196	0.009
Supervisory Attentional System	←	Conscious Regulation of Emotion	0.121	0.046	0.120	0.009
Management of Elements to Solve Tasks	←	Behavioral Verification for Learning	0.221	0.052	0.232	0.001
Decision-Making	←	Conscious Regulation of Behavior	0.466	0.109	0.353	0.001
Management of Elements to Solve Tasks	←	Conscious Regulation of Behavior	0.861	0.111	0.527	0.001
Decision-Making	←	Conscious Regulation of Emotion	0.204	0.032	0.314	0.001
Decision-Making	←	Supervisory Attentional System	0.149	0.037	0.231	0.001

*, Simple Linear Regression; *B*, Unstandardized coefficient; S.E., Standard error; *β*, Standardized coefficient.

For the Supervisory Attentional System, the structural equation model showed acceptable fit indices: *χ*^2^(147) = 883.091, *p* < 0.001; RMSEA = 0.06, 90% CI [0.060, 0.068], *p* < 0.001; CFI = 0.89; SRMR = 0.04. This variable received significant influences from Conscious Monitoring of Responsibilities (*β* = 0.474, *B* = 0.678, *p* < 0.001) and Conscious Regulation of Behavior (*β* = 0.354, *B* = 0.659, *p* < 0.001). However, Decision-Making did not show a significant effect on this executive function (*β* = 0.077, *B* = 0.121, *p* = 0.206). Likewise, Decision-Making had a positive effect on Conscious Regulation of Behavior (*β* = 0.767, *B* = 0.646, *p* < 0.001), and Conscious Regulation of Behavior, in turn, influenced Conscious Monitoring of Responsibilities (*β* = 0.675, *B* = 0.878, *p* < 0.001) (see Table 3).

The structural equation model for Conscious Regulation of Behavior showed an excellent level of fit based on the following values: *χ*^2^(87) = 550.089, *p* < 0.001; RMSEA = 0.06, 90% CI [0.06, 0.07], *p* < 0.001; CFI = 0.90; SRMR = 0.04. All unstandardized regression weights indicated significant relationships among the factors studied (see Table 3). First, Conscious Monitoring of Responsibilities had a positive and significant effect on Conscious Regulation of Emotion (*β* = 0.330, *B* = 0.482, *p* < 0.001). Likewise, Conscious Monitoring of Responsibilities also had a positive effect on Conscious Regulation of Behavior (*β* = 0.430, *B* = 0.440, *p* < 0.001). In addition, Conscious Regulation of Emotion was positively associated with Conscious Regulation of Behavior (*β* = 0.540, *B* = 0.382, *p* < 0.001).

For Behavioral Verification for Learning, the structural equation model showed acceptable fit indices: *χ*^2^(74) = 634.667, *p* < 0.001; RMSEA = 0.07, 90% CI [0.07, 0.08], *p* < 0.001; CFI = 0.91; SRMR = 0.05. This function was positively explained by the Supervisory Attentional System (*β* = 0.354, *B* = 0.366, *p* < 0.001) and Conscious Monitoring of Responsibilities (*β* = 0.714, *B* = 0.543, *p* < 0.001). Likewise, the Supervisory Attentional System was predicted by Conscious Monitoring of Responsibilities with a very high coefficient (*β* = 0.765, *B* = 0.972, *p* < 0.001) (see Table 3).

Regarding Decision-Making, the structural equation model showed acceptable fit indices: *χ*^2^(130) = 934.346, *p* < 0.001; RMSEA = 0.07, 90% CI [0.06, 0.07], *p* < 0.001; CFI = 0.89; SRMR = 0.06. This function was positively predicted by the Supervisory Attentional System (*β* = 0.308, *B* = 0.213, *p* < 0.001), Conscious Regulation of Behavior (*β* = 0.300, *B* = 0.324, *p* < 0.001), and Conscious Regulation of Emotion (*β* = 0.302, *B* = 0.193, *p* < 0.001). In turn, Conscious Regulation of Emotion had a strong effect on the Supervisory Attentional System (*β* = 0.542, *B* = 0.501, *p* < 0.001), and Conscious Regulation of Behavior influenced Conscious Regulation of Emotion with a high coefficient (*β* = 0.734, *B* = 1.238, *p* < 0.001) (see Table 3).

Conscious Regulation of Emotion represented a particular case, as in the decision tree it was predicted only by Conscious Regulation of Behavior. Because the interaction involved only two variables, a simple linear regression was conducted, demonstrating its influence (*β* = 0.489, *B* = 0.512, *p* < 0.001) (see Table 3).

The structural equation model for Management of Elements to Solve Tasks showed acceptable fit indices: *χ*^2^(74) = 372.628, *p* < 0.001; RMSEA = 0.05, 90% CI [0.05, 0.06], *p* < 0.001; CFI = 0.92; SRMR = 0.04. The results showed that this function received a positive and significant effect from Behavioral Verification for Learning (*β* = 0.208, *B* = 0.167, *p* < 0.001) and, to a greater extent, from Conscious Regulation of Behavior (*β* = 0.521, *B* = 0.823, *p* < 0.001). In addition, Conscious Regulation of Behavior was influenced by Behavioral Verification for Learning (*β* = 0.028, *B* = 0.312, *p* < 0.001) (see Table 3).

### 3.3 Construction of the unified explanatory model

The initial specification of the paths in the unified explanatory model was based on the convergence of three sources of evidence: the results of the decision-tree analyses, the coefficients from the independent SEM models, and theoretical coherence with models of executive control. The decision trees recurrently identified Conscious Monitoring of Responsibilities and Behavioral Verification for Learning as main predictors of multiple executive functions, whereas Conscious Regulation of Emotion, Conscious Regulation of Behavior, and the Supervisory Attentional System emerged as key mediators, and Decision-Making and Management of Elements to Solve Tasks appeared as final dependent variables (see Table 3).

The independent SEM models confirmed the magnitude and significance of these relationships. Conscious Monitoring of Responsibilities significantly predicted Conscious Regulation of Emotion (*β* = 0.330, *p* < 0.001), Conscious Regulation of Behavior (*β* = 0.430, *p* < 0.001), and the Supervisory Attentional System (*β* = 0.474/0.765, *p* < 0.001). Behavioral Verification for Learning predicted the Supervisory Attentional System (*β* = 0.670, *p* < 0.001) and Management of Elements to Solve Tasks (*β* = 0.208, *p* < 0.001). Conscious Regulation of Emotion predicted Conscious Regulation of Behavior (*β* = 0.540, *p* < 0.001), the Supervisory Attentional System (*β* = 0.542, *p* < 0.001), and Decision-Making (*β* = 0.302, *p* < 0.001). Conscious Regulation of Behavior predicted the Supervisory Attentional System (*β* = 0.354, *p* < 0.001), Decision-Making (*β* = 0.300, *p* < 0.001), and Management of Elements to Solve Tasks (*β* = 0.521, *p* < 0.001). Finally, the Supervisory Attentional System predicted Decision-Making (*β* = 0.308, *p* < 0.001).

The inclusion of the path Supervisory Attentional System ← Decision-Making was discarded because it did not reach statistical significance in the corresponding SEM model (*β* = 0.077, *p* = 0.206). The resulting hierarchical structure enabled the elimination of the feedback loops identified in the decision trees while preserving all substantive relationships, in accordance with models of executive architecture that describe a directional flow from metacognition to regulation, attentional allocation, and action (Barkley, 2012; Diamond, 2013).

#### 3.3.1 Model fit

The initial unified model showed a moderate fit, indicating the need for refinement: *χ*^2^(418) = 2071.540, *p* < 0.001; CFI = 0.88; RMSEA = 0.057, 90% CI [0.054, 0.059]; SRMR = 0.05. First, the significance of the structural regression coefficients was examined. The path Conscious Regulation of Emotion ← Behavioral Verification for Learning did not reach statistical significance, suggesting that this relationship may be fully mediated by other variables within the system. The path Management of Elements to Solve Tasks ← Supervisory Attentional System was also removed due to non-significance. These eliminations improved the model’s parsimony (PRATIO = 0.899) without compromising its explanatory power.

In addition, an analysis of the Modification Indices (MI) was conducted. This analysis identified error covariances between items with values above 10 that were theoretically justifiable. Fourteen error covariances were added and organized into two categories according to their theoretical rationale. Covariances within the same executive function, interpreted as method effects, reflected highly similar wording and content among items assessing closely related facets of the same construct. In Behavioral Verification for Learning, Item 20, “I check that my university assignments are well done and error-free before submitting them to the professor,” and Item 23, “I review the spelling and writing of my university assignments before finalizing them” (MI = 52.86), as well as Items 23 and 30, “When I finish a university activity, I check whether I have achieved what I had planned” (MI = 16.13), shared the component of formal review of written assignments.

In Conscious Regulation of Behavior, Item 3, “I always act by thinking and reflecting on the consequences of my actions,” and Item 19, “I can anticipate the consequences of my actions,” converged in the assessment of the ability to anticipate consequences (MI = 44.24). In addition, Item 16, “It is easy for me to behave appropriately in social gatherings,” and Item 18, “I let others speak without interrupting,” shared a component related to behavioral modulation and adjustment to social norms (MI = 19.961).

In the Supervisory Attentional System, Item 10, “I have good concentration,” and Item 14, “I am able to maintain attention on an activity,” both referred to sustained attentional capacity (MI = 19.61). In Management of Elements to Solve Tasks, Item 7, “It is easy for me to quickly locate my materials when I look for them in my room or at my desk,” and Item 6, “I keep my things in the right place and in order” (MI = 13.31), as well as Items 7 and 1, “It is easy for me to pick up and leave my things organized when I am asked to do so” (MI = 10.12), shared the component of organization and accessibility of the material environment. Finally, in Conscious Monitoring of Responsibilities, Item 15, “I can do my work without someone supervising me,” and Item 8, “I can complete university tasks independently and without help from others,” shared the core element of autonomy in academic task execution (MI = 124.29).

Covariances between items belonging to different executive functions, interpreted as inter-construct semantic overlap, captured the conceptual interdependence among executive functions. The strongest covariance was observed between Item 11 of Conscious Regulation of Behavior, “I can remain still and calm while waiting,” and Item 25 of Conscious Regulation of Emotion, “I remain calm easily” (MI = 62.01). Similarly, Item 3 of Conscious Regulation of Behavior, “I always act by thinking and reflecting on the consequences of my actions,” and Item 4 of Conscious Regulation of Emotion, “I regulate my emotions appropriately,” represented two manifestations of the same executive control mechanism (MI = 35.73). Furthermore, Item 28 of the Supervisory Attentional System, “I maintain good study habits,” and Item 27 of Conscious Monitoring of Responsibilities, “I finish my university assignments on time,” showed a substantial covariance (MI = 38.25), indicating that both are interrelated behavioral expressions of the same continuum of academic self-regulation. Likewise, Item 28 of the Supervisory Attentional System and Item 24 of Behavioral Verification for Learning, “I remember to bring home university tasks, materials, or papers,” covaried significantly (MI = 39.07), as both share an underlying component of systematicity and consistency in the university context.

#### 3.3.2 Measurement invariance analysis by sex

Table 4 presents the fit indices for the three levels of invariance tested sequentially. The configural model showed acceptable fit (CFI = 0.905, RMSEA = 0.036), indicating that the underlying factor structure (the seven factors and their relationships with the items) is the same in men and women. The metric model exhibited fit practically identical to the configural model (CFI = 0.904, ΔCFI = −0.001; RMSEA = 0.036, ΔRMSEA = 0.000), demonstrating that factor loadings are equivalent across sexes. This implies that each item contributes with the same magnitude to the measurement of its respective executive function in both groups.

**Table 4:** Fit indices for the measurement invariance models by sex.

Model	*χ* ^2^	df	RMSEA	IC 90% RMSEA	CFI	ΔCFI	ΔRMSEA
Configural	2,117.486	814	0.036	[0.034, 0.038]	0.905	–	–
Metric	2,141.487	827	0.036	[0.034, 0.038]	0.904	−0.001	0.000
Scalar	2,242.631	858	0.039	[0.037, 0.041]	0.884	−0.020	0.003

However, the scalar model showed a significant deterioration in fit (CFI = 0.884, ΔCFI = −0.020; RMSEA = 0.039, ΔRMSEA = 0.003). The drop in CFI exceeded the critical threshold of −0.01, indicating that item intercepts differ between men and women. In substantive terms, this means that individuals with the same latent level of an executive function tend to obtain systematically different observed scores on some specific items, depending on their sex. It is important to emphasize that configural and metric invariance are indeed satisfactorily met, which guarantees that the hierarchical structure of the explanatory model and the predictive relationships among executive functions are invariant across sexes. Therefore, the conclusions proposed regarding the organization of executive functions are applicable to both men and women.

#### 3.3.3 Bootstrapping analysis

The bootstrap discrepancy distribution had a mean of 2128.855 (S.E. = 2.414), indicating acceptable variability in model fit across replications. The 90% confidence intervals (percentile and bias-corrected) for the structural paths of the final model did not include zero in any case, confirming the statistical significance of all structural coefficients (see Table 5).

**Table 5:** Bootstrap confidence intervals (percentile and bias-corrected) for the standardized structural paths of the final model.

Paths	*β*	CI 90% Percentile	CI 90% bias-corrected	*p*
Level 1 → Level 2				
Conscious Monitoring of Responsibilities → Conscious Regulation of Emotions	0.358	[0.297, 0.416]	[0.297, 0.416]	0.001
Conscious Monitoring of Responsibilities → Conscious Regulation of Behavior	0.197	[0.004, 0.369]	[0.004, 0.369]	0.092
Behavior Verification for Learning → Conscious Regulation of Behavior	0.380	[0.220, 0.552]	[0.220, 0.552]	0.001
Level 1 → Level 1				
Conscious Regulation of Emotions → Conscious Regulation of Behavior	0.494	[0.429, 0.559]	[0.429, 0.559]	0.001
Level 2 → Level 3				
Behavior Verification for Learning → Supervisory Attention System	0.214	[0.056, 0.347]	[0.056, 0.347]	0.042
Conscious Monitoring of Responsibilities → Supervisory Attention System	0.445	[0.320, 0.572]	[0.320, 0.572]	0.001
Conscious Regulation of Behavior → Supervisory Attention System	0.196	[0.040, 0.379]	[0.040, 0.379]	0.034
Conscious Regulation of Emotions → Supervisory Attention System	0.120	[0.010, 0.212]	[0.010, 0.212]	0.068
Level 3 → Level 4				
Conscious Regulation of Behavior → Decision-Making	0.353	[0.148, 0.561]	[0.148, 0.561]	0.012
Conscious Regulation of Emotions → Decision-Making	0.314	[0.194, 0.423]	[0.194, 0.423]	0.001
Supervisory Attention System → Decision-Making	0.231	[0.084, 0.368]	[0.084, 0.368]	0.010
Behavior Verification for Learning → Management of Elements to Solve Tasks	0.232	[0.118, 0.349]	[0.118, 0.349]	0.002
Conscious Regulation of Behavior → Management of Elements to Solve Tasks	0.527	[0.410, 0.636]	[0.410, 0.636]	0.001

*β*, Standardized coefficient, CI, Confidence interval.

#### 3.3.4 The hierarchical scaffolding model of executive functioning in Cuban university students

**Figure 1:**
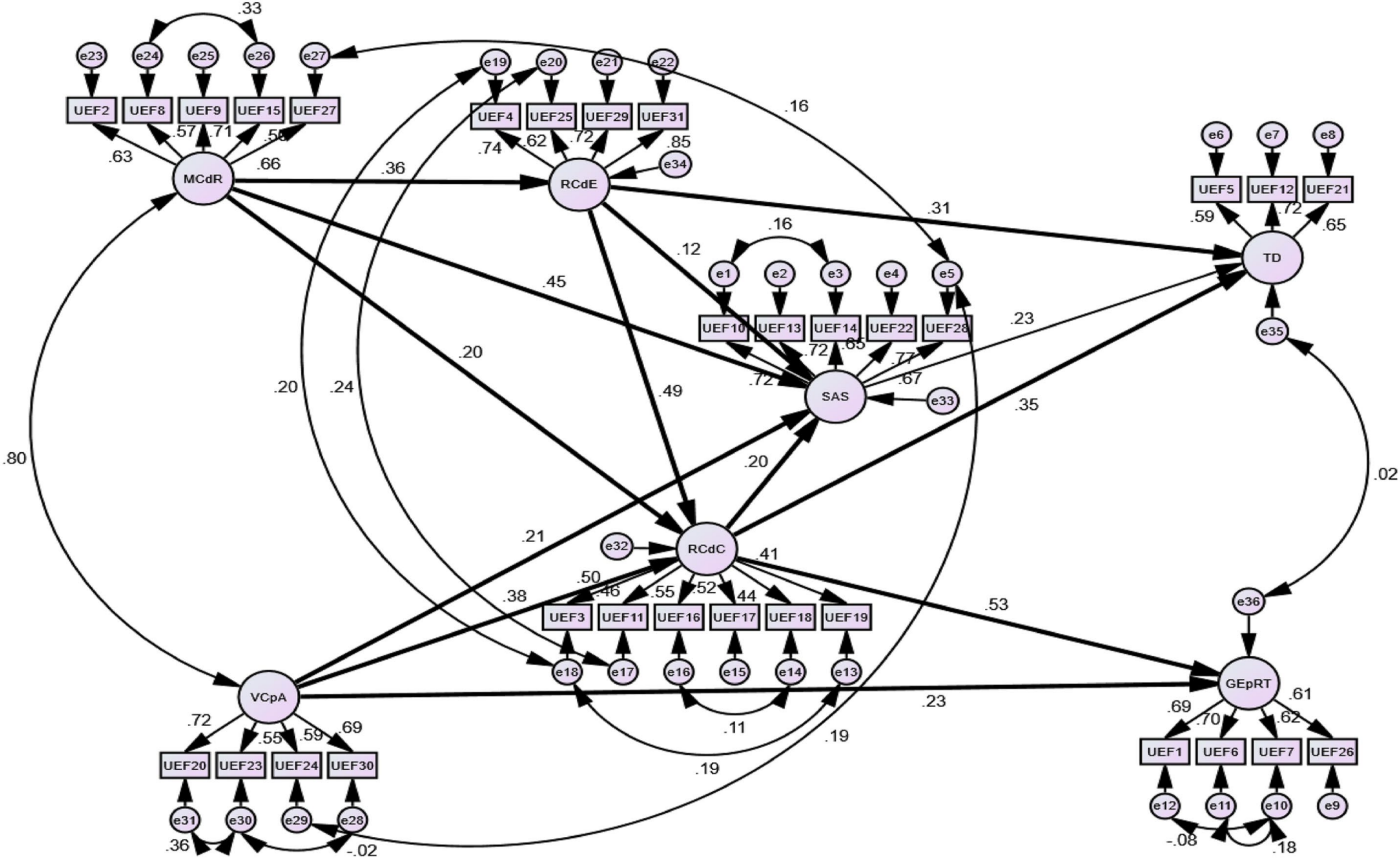
Structural equation model for the unified explanatory model. MCdR, Conscious monitoring of responsibilities; SAS, Supervisory attention system; RCdC, Conscious regulation of behavior; VCpA, Behavior verification for learning; TD, Decision-making; RCdE, Conscious regulation of emotions; GEpRT, Management of elements to solve tasks. The authors created this figure using IBM SPSS Amos v.22.0, no third-party copyrighted material has been used.

**Figure 2:**
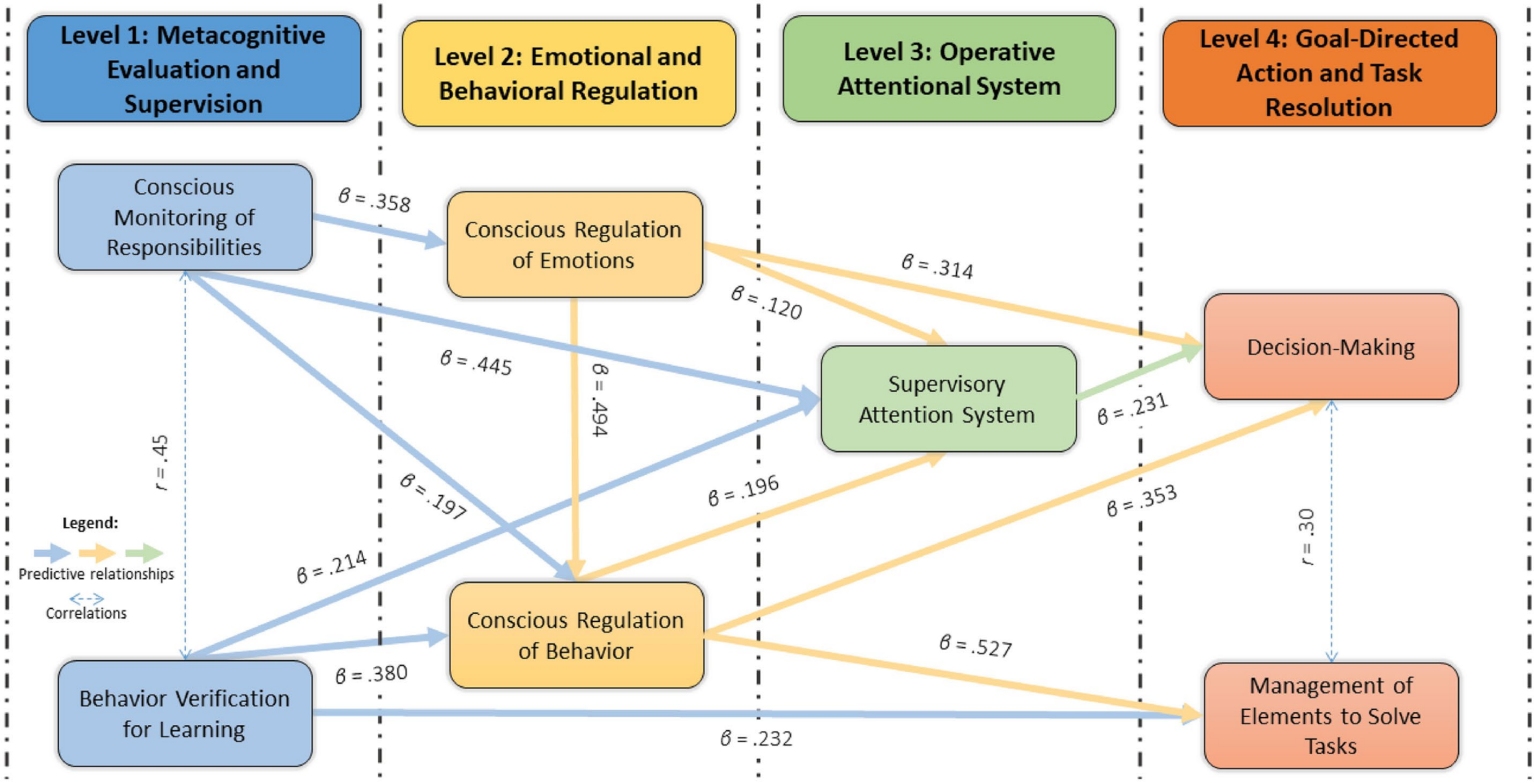
Graphical representation of the explanatory model of executive functions in Cuban university students. The authors created this figure using Microsoft PowerPoint, no third-party copyrighted material has been used.

The resulting model showed satisfactory fit to the data: *χ*^2^(407) = 1580.914, *p* < 0.001; CFI = 0.915; RMSEA = 0.048, 90% CI [0.046, 0.051]; SRMR = 0.045 (see Figure 1). The improvement relative to the initial model was substantial (ΔCFI = +0.035, ΔRMSEA = −0.009) and was achieved without compromising parsimony (PRATIO = 0.875, PNFI = 0.778, PCFI = 0.801) or altering the substantive structure of the model (see Table 2). The graphical representation of the model is presented in Figure 2.

Table 6 presents the decomposition of standardized effects (*β*) and coefficients of determination (*R*^2^) for each endogenous variable of the final explanatory model. Conscious Regulation of Emotion (F6, *R*^2^ = 0.128) is explained exclusively by a direct effect of Conscious Monitoring of Responsibilities (F1 → F6, *β* = 0.358), with no indirect effects. The absence of other predictors indicates that, in the model, conscious emotional regulation is primarily associated with awareness of academic responsibilities.

**Table 6:** Standardized direct, indirect, and total effects (*β*) and coefficients of determination (*R*^2^).

Factors	Effect (*β*)	F1	F4	F6	F3	F2	F7	F5
F6 (*R*^2^ = 0.128)	Indirect	0.000	0.000	0.000	0.000	0.000	0.000	0.000
	Direct	0.358	0.000	0.000	0.000	0.000	0.000	0.000
	Total	0.358	0.000	0.000	0.000	0.000	0.000	0.000
F3 (*R*^2^ = 0.724)	Indirect	0.177	0.000	0.000	0.000	0.000	0.000	0.000
	Direct	0.197	0.380	0.494	0.000	0.000	0.000	0.000
	Total	0.374	0.380	0.494	0.000	0.000	0.000	0.000
F2 (*R*^2^ = 0.710)	Indirect	0.116	0.075	0.097	0.000	0.000	0.000	0.000
	Direct	0.445	0.214	0.120	0.196	0.000	0.000	0.000
	Total	0.562	0.289	0.217	0.196	0.000	0.000	0.000
F7 (*R*^2^ = 0.497)	Indirect	0.197	0.200	0.260	0.000	0.000	0.000	0.000
	Direct	0.000	0.232	0.000	0.527	0.000	0.000	0.000
	Total	0.197	0.432	0.260	0.527	0.000	0.000	0.000
F5 (*R*^2^ = 0.613)	Indirect	0.374	0.201	0.225	0.045	0.000	0.000	0.000
	Direct	0.000	0.000	0.314	0.353	0.231	0.000	0.000
	Total	0.374	0.201	0.539	0.398	0.231	0.000	0.000

Conscious Monitoring of Responsibilities (F1), Supervisory Attention System (F2), Conscious Regulation of Behavior (F3), Behavior Verification for Learning (F4), Decision-Making (F5), Conscious Regulation of Emotions (F6), Management of Elements to Solve Tasks (F7). All reported effects are statistically significant (*p* < 0.05), except where a value of 0.000 is indicated (absence of effect).

Conscious Regulation of Behavior (F3, *R*^2^ = 0.724) showed the highest coefficient of determination in the model, indicating that three predictors jointly explain 72.4% of its variance. The strongest direct effects come from Conscious Regulation of Emotion (F6 → F3, *β* = 0.494) and Behavioral Verification for Learning (F4 → F3, *β* = 0.380), while Conscious Monitoring of Responsibilities (F1 → F3) contributes a more modest but significant direct effect (*β* = 0.197), in addition to a relevant indirect effect (*β* = 0.177, through F6). This configuration positions behavioral regulation as a critical integration node, receiving convergent influences from metacognition, learning verification, and emotional regulation.

The Supervisory Attentional System (F2, *R*^2^ = 0.710) receives influences from four predictors, with total effects ranging from 0.196 (from F3) to 0.562 (from F1). Notably, the strongest total effect comes from Conscious Monitoring of Responsibilities (F1 → F2, *β* total = 0.562), which consists of a substantial direct effect (*β* = 0.445) and a moderate indirect effect (*β* = 0.116, mediated by F6 and F3). Behavioral Verification for Learning (F4) exerts a relevant total effect (*β* = 0.289), combining direct (*β* = 0.214) and indirect (*β* = 0.075) influences through F3. Notably, Conscious Regulation of Emotion (F6) exerts its influence on F2 both directly (*β* = 0.120) and indirectly through F3 (*β* = 0.097), suggesting that emotional stability is structurally positioned prior to attention and also operates through behavioral regulation.

Management of Elements to Solve Tasks (F7, *R*^2^ = 0.497) receives its strongest influence from Conscious Regulation of Behavior (F3 → F7, *β* direct = 0.527), suggesting that the organization of the material environment depends on the capacity to regulate behavior. Behavioral Verification for Learning (F4) exerts a total effect of 0.432, composed of a direct effect (*β* = 0.232) and a relevant indirect effect (*β* = 0.200) through F3. It is noteworthy that Conscious Monitoring of Responsibilities (F1) and Conscious Regulation of Emotion (F6) influence F7 exclusively indirectly (*β* total = 0.197 and 0.260, respectively), with no direct effects. This pattern shows that higher-level metacognitive and emotional processes do not translate directly into environmental organization, but rather require the mediation of behavioral regulation and learning verification.

Decision-Making (F5, *R*^2^ = 0.613) shows a notably complex pattern of effects. The predictor with the largest total effect on F5 is Conscious Regulation of Emotion (F6 → F5, *β* total = 0.539), which exerts a mixed influence: a considerable direct effect (*β* = 0.314) and a substantial indirect effect (*β* = 0.225) mediated by F3 and F2. This configuration suggests that emotional stability contributes to better decision-making both directly and through the strengthening of behavioral and attentional regulation. Conscious Regulation of Behavior (F3 → F5, *β* total = 0.398) and Conscious Monitoring of Responsibilities (F1 → F5, *β* total = 0.374) also exert relevant total effects, mostly indirect in the case of F1 (*β* indirect = 0.374) and mixed in the case of F3 (*β* direct = 0.353, *β* indirect = 0.045). Finally, the Supervisory Attentional System (F2) contributes a moderate direct effect (*β* = 0.231), pointing to the fact that attentional capacity is a necessary resource for weighing alternatives in decision-making contexts.

The structure of the model (see Figures 1, 2) enables understanding of the proposed interrelations that executive functions show as they converge on an academic demand. This configuration describes patterns of interrelation rather than an invariable sequence and may be modulated by the nature of the task, the student’s learning history, and the resources available in the environment.

In the following sections, we describe each level of the model. First, we present the empirical findings. Then, we offer theoretical interpretations of these patterns, which are intended to enrich the conceptual understanding of the model and to generate hypotheses for future research. These interpretations are proposed as plausible conceptual extensions of the empirical findings, rather than as conclusions directly demonstrated by the data.

##### 3.3.4.1 Level 1: metacognitive evaluation and supervision

This level represents the moment when the student’s subjective experience is reorganized around a demand that takes on personal significance. It is therefore a process of conscious mediation through which an academic obligation is interpreted, evaluated, and transformed into a sufficiently meaningful motive to become articulated as a need. Within the model, the sequence is represented as beginning when the student perceives an academic obligation, which is captured by Conscious Monitoring of Responsibilities. This function operates as a constantly active metacognitive identifier and exerts direct effects on three processes at the immediately lower level: Conscious Regulation of Emotion (*B* = 0.464, *β* = 0.358, *p* < 0.001), Conscious Regulation of Behavior (*B* = 0.126, *β* = 0.197, *p* = 0.008), and the Supervisory Attentional System (*B* = 0.581, *β* = 0.445, *p* < 0.001). In parallel, Behavioral Verification for Learning is structurally linked to this process by reviewing the status of already completed tasks, exerting direct effects on Conscious Regulation of Behavior (*B* = 0.222, *β* = 0.380, *p* < 0.001), the Supervisory Attentional System (*B* = 0.256, *β* = 0.214, *p* = 0.002), and Management of Elements to Solve Tasks (*B* = 0.221, *β* = 0.232, *p* < 0.001). Both functions, correlated at the upper level of the model, jointly are represented as contributing to the metacognitive signal that is associated with the rest of the executive system.

In the model, Conscious Monitoring of Responsibilities and Behavioral Verification for Learning operate as psychological tools of social origin that the student has internalized throughout their educational trajectory and that allow them to regulate their own activity without relying exclusively on immediate external contingencies. This internalization transforms the academic requirement, originally external and derived from the curriculum, the teacher, or the institutional environment, into a need that the student recognizes as their own. When this level operates effectively, awareness of responsibility is experienced as mastery over one’s own activity, which is associated with the subjective conditions that, in the proposed model, are associated with the functioning of the rest of the system.

##### 3.3.4.2 Level 2: emotional and behavioral regulation

This level describes the transition from emotional experience to controlled action, a process that involves the student’s ability to establish a pause between impulse and response. In this sense, once the student becomes aware of their needs and responsibilities, a process of Conscious Regulation of Emotion begins. Conscious Monitoring of Responsibilities is associated with this regulation (*B* = 0.464, *β* = 0.358, *p* < 0.001), suggesting that awareness of academic obligations acts as a support mechanism that promotes emotional stability. This Conscious Regulation of Emotion is represented as a protective filter that is associated with system functionality, and this stability, in the model, is structurally linked to the conditions under which Conscious Regulation of Behavior operates. Through this function, the student inhibits impulsive responses, reflects on the consequences of their actions, and adjusts their behavior to the demands of the situation. This function simultaneously receives inputs from three sources: Conscious Monitoring of Responsibilities (*B* = 0.126, *β* = 0.197, *p* = 0.008), Conscious Regulation of Emotion (*B* = 0.243, *β* = 0.494, *p* < 0.001), and Behavioral Verification for Learning (*B* = 0.222, *β* = 0.380, *p* < 0.001). This level is critical because all subsequent executive functions are structurally related, directly or indirectly, on Conscious Regulation of Behavior: it is associated with the Supervisory Attentional System (*B* = 0.402, *β* = 0.196, *p* = 0.009), Decision-Making (*B* = 0.466, *β* = 0.353, *p* < 0.001), and Management of Elements to Solve Tasks (*B* = 0.861, *β* = 0.527, *p* < 0.001).

From a psychological development perspective, the emotional and behavioral modulation proposed at this level has a social origin. Strategies for Conscious Regulation of Emotion are initially learned through interaction with significant others and are progressively internalized as forms of self-governance. Once emotional stability is achieved, the student can engage in Conscious Regulation of Behavior, which involves inhibiting impulsive responses, anticipating consequences, and deliberately adjusting behavior. In this sense, neither higher-order process is assumed to emerge spontaneously or solely as a result of maturation; rather, both arise from the internalization of socially organized forms of behavior. Only after regulation has first been experienced at the interpsychological, or social, level can the individual deploy it at the intrapsychological level as a personal resource.

##### 3.3.4.3 Level 3: operational attentional system

This level represents the point at which subjective resources are most strongly concentrated on the task. The Supervisory Attentional System is configured as a receiving and integrative node that receives direct inputs from four different sources, reflecting its nature as a limited-capacity resource supported by multiple executive processes. Conscious Regulation of Behavior contributes to this system (*B* = 0.402, *β* = 0.196, *p* = 0.009), as the ability to inhibit external and internal distractors helps maintain focus on the task. In addition, it receives direct inputs from Conscious Monitoring of Responsibilities (*B* = 0.581, *β* = 0.445, *p* < 0.001), Behavioral Verification for Learning (*B* = 0.256, *β* = 0.214, *p* = 0.002), and Conscious Regulation of Emotion (*B* = 0.121, *β* = 0.120, *p* = 0.009). Although modest in magnitude, this latter relationship indicates that Conscious Regulation of Emotion frees attentional resources that become available for task supervision.

Therefore, the Supervisory Attentional System is not represented as operating through a single pathway; rather, it integrates information derived from metacognition, verification, emotional regulation, and behavioral regulation to enhance the capacity for sustained work. In this sense, the model conceptualizes supervisory attention as a capacity sustained by the internalization of socially available resources, such as schedules, reminders, and socially acquired habits, and mediated by the subjective meaning that the task holds for the student based on their system of norms and values.

##### 3.3.4.4 Level 4: goal-directed action and task resolution

This level marks the moment when executive control is materialized in socially meaningful behaviors that transform the student’s relationship with the academic environment. The model identifies two types of executive outputs that constitute visible academic action. Decision-Making is represented as being initiated when the student must choose among alternatives and receives inputs from three sources: Conscious Regulation of Behavior (*B* = 0.466, *β* = 0.353, *p* < 0.001), Conscious Regulation of Emotion (*B* = 0.204, *β* = 0.314, *p* < 0.001), and the Supervisory Attentional System (*B* = 0.149, *β* = 0.231, *p* < 0.001). This configuration indicates that the quality of academic decisions does not depend exclusively on the capacity to reflect and inhibit impulses, but also on the emotional stability with which the student approaches the choice and on the attentional resources they can mobilize to evaluate alternatives. In turn, Management of Elements to Solve Tasks is represented as being initiated when the student needs to organize their material and temporal environment in order to act. This function receives inputs from both Conscious Regulation of Behavior (*B* = 0.861, *β* = 0.527, *p* < 0.001), its strongest predictor, and Behavioral Verification for Learning (*B* = 0.221, *β* = 0.232, *p* < 0.001), suggesting that organization of the study environment benefits from both general behavioral control and the specific habit of verifying and reviewing academic tasks.

On this basis, Decision-Making is proposed not merely as a process of rational choice among alternatives, but as a function supported by previously mobilized emotional stability, behavioral control, and attentional resources. This allows choice to be articulated as the conscious-volitional mobilization of personal psychological resources toward the fulfillment of a need, rather than as an immediate reaction to situational pressure. Management of Elements to Solve Tasks, in turn, refers to the material and contextual dimension of executive functioning: organizing the environment, arranging resources, and sequencing actions as forms of instrumental mediation through which the student structures their space to sustain goal-directed activity. Both operational outputs represent the culmination of a self-regulatory process that, although manifested in observable behaviors, originates in the internalization of social relationships and in the appropriation of cultural tools that allow the student to adapt to academic demands and actively configure the conditions of their own learning.

## 4 Discussion

The central objective of this study was to design an explanatory model of executive functioning in Cuban university students. Before discussing the implications of the model, it is important to clarify the nature of the construct being modeled. The present study relies on self-report measures (UEF-1), which assess students’ perceptions of their executive functioning in academic contexts. According to Barkley's (2012) extended phenotype model, such measures evaluate higher-order levels of the executive system (specifically the Methodical–Autonomous, Tactical–Reciprocal, and Strategic–Cooperative levels) which are more closely associated with real-world adaptive functioning and impairments in major life activities than are performance-based tests. Performance-based tests, in contrast, primarily assess the Instrumental–Self-Directed level, capturing cognitive processes in controlled, short-term contexts (Barkley, 2012; Toplak et al., 2013).

Consistent with this framework, the model proposed here reflects the perceived organization of executive functions at these higher phenotypic levels, as they manifest in the daily academic lives of university students. This does not diminish the value of the findings; rather, it situates them within a broader understanding of executive functioning as a multilevel, ecologically embedded construct. The hierarchical architecture observed represents a plausible organization of self-perceived executive capacities that may be particularly relevant for understanding academic self-regulation and for designing interventions aimed at strengthening these capacities in educational contexts. Future studies combining self-report with performance-based measures and observer reports would be valuable to examine the convergence and divergence across different levels of the extended phenotype.

Based on our results, the proposed model presents executive functions as organized into four sequential levels (Metacognitive Evaluation and Supervision, Emotional and Behavioral Regulation, Operational Attentional System, and Goal-Directed Action and Task Resolution) and suggests a sequential flow from higher-order metacognitive processes toward operational output processes in a sequential flow (see Figures 1, 2). It is important to note that the proposed model represents a plausible organization of the perceived executive system, rather than a definitive demonstration of causality. The hierarchical architecture and the directionality of influences are empirically derived from the data and theoretically interpreted, but they should be understood as testable hypotheses that require confirmation through experimental, longitudinal, or intervention studies. With this caveat in mind, we discuss the main findings and their implications.

Regarding the levels obtained for executive functions, it was observed that functions at Level 4, such as Decision-Making and Management of Elements to Solve Tasks, were notably above average. This could reflect skills that are particularly stimulated by the university academic context in relation to operational output processes (Valdes and Lopez, 2024; Wang et al., 2022). Conversely, areas of relative weakness were identified in functions located at Levels 1, 2, and 3 of the model: Conscious Monitoring of Responsibilities, Conscious Regulation of Behavior, and the Supervisory Attentional System, consistent with widely documented challenges in student self-regulation (Nota et al., 2004).

This diagnostic profile provided the starting point for examining how these capacities are related to one another. The proposed explanatory model shows that the executive system is organized into four levels, with a predominant directionality from metacognition toward action emerging from the data.

At Level 1, Metacognitive Evaluation and Supervision, Conscious Monitoring of Responsibilities and Behavioral Verification for Learning are correlated exogenous variables that are statistically associated with the functioning of the rest of the system in the proposed model. This dual configuration of metacognition, involving monitoring and verification, is consistent with models such as those proposed by Frazier et al. (2021) and Gambo and Shakir (2021), which suggest that self-regulation begins with processes of self-observation and self-evaluation that are linked to the rest of the system. The fact that both functions are exogenous and correlated suggests that there is no causal precedence between monitoring responsibilities and verifying learning; rather, they constitute two facets of the same metacognitive process that mutually reinforce each other. As Lebuda and Benedek (2023) point out, metacognition is a multifaceted process.

At Level 2, Emotional and Behavioral Regulation, the model shows a sequential organization in which Conscious Regulation of Emotion functionally demonstrates a structural influence on Conscious Behavior Regulation, consistent with the theoretically specified directionality. The former is associated with Conscious Monitoring of Responsibilities, suggesting that awareness of academic obligations may acts as a support mechanism that promotes emotional stability. Conscious Regulation of Emotion appears, in the model, as a protective filter that is structurally linked to the conditions under which Conscious Regulation of Behavior operates, showing a substantial association with it. In this sense, the present study contributes to a body of knowledge that, while recognizing the bidirectionality between emotion and behavior, offers evidence consistent with the hypothesis that behavioral regulation, as a higher mental function, is complex in its origin because it does not emerge directly from instruction about “how to behave.” Rather, it is supported by previous experiences of emotional regulation that have already been internalized (Halse et al., 2024; Murray et al., 2022). However, this directional interpretation should be confirmed through longitudinal research.

Level 3, the Operational Attentional System, is configured as a receiving and integrative node that receives direct inputs from four different sources: Conscious Monitoring of Responsibilities, Behavioral Verification for Learning, Conscious Regulation of Behavior, and Conscious Regulation of Emotion. This reflects its nature as a limited-capacity resource that, in the proposed model, is statistically associated with multiple executive processes. The configuration of the Supervisory Attentional System as an integrative node receiving input from four sources is consistent with the classic model of Norman and Shallice (1986) and with Wickens’ (2021) conceptualizations, in which the supervisory attentional system does not operate in isolation but receives information from multiple subsystems in order to allocate resources to non-routine tasks.

The proposed model specifies what these sources are within the academic context and quantifies their relative contribution, revealing that Conscious Monitoring of Responsibilities is the strongest predictor of the Supervisory Attentional System.

This finding suggests that, within the proposed model, sustained attention is statistically associated with the clarity with which students perceive they have defined their responsibilities and with the emotional and behavioral stability they have previously developed. As Fournier et al. (2026) and Sun et al. (2026) note, anxiety and emotional dysregulation consume resources from working memory and the attentional system. Thus, the model suggests that emotional regulation is structurally related to attention in a way that may free attentional capacity that would otherwise be occupied by worry and distress.

Level 4, Goal-Directed Action and Task Resolution, is represented as producing the operational outputs of the system. Decision-Making integrates influences from three sources: Conscious Regulation of Behavior, Conscious Regulation of Emotion, and the Supervisory Attentional System. This configuration suggests that the quality of academic decisions may be associated with not exclusively on the capacity to reflect and inhibit impulses, but also on the emotional stability with which the student approaches the choice and on the attentional resources they are able to mobilize to evaluate alternatives (Sun et al., 2026; Rad et al., 2025). Management of Elements to Solve Tasks, in turn, receives inputs from Conscious Regulation of Behavior and Behavioral Verification for Learning.

The differentiation between two operational outputs, one oriented toward choosing and the other toward organizing, constitutes a specific contribution of the model. Whereas Decision-Making integrates emotional, behavioral, and attentional influences, reflecting its nature as a process that requires weighing alternatives under conditions of uncertainty (Cristofaro, 2020; Raouf and Abbes, 2025), Management of Elements to Solve Tasks is more closely linked to behavioral control and the habit of verification (Susanti, 2021). This is consistent with the idea that organizing the material and temporal environment is a competence strengthened through the systematic practice of reviewing and adjusting working conditions. As Kim et al. (2023) argue, self-regulation does not culminate in internal self-control, but in the capacity to modify the environment so that it serves the individual’s goals, transforming the context into an ally of executive functioning.

Within the model, executive functions do not operate on a single plane of reciprocal relationships. Instead, they are organized in a sequence of functional precedence, in which processes at one level are associated with those at the immediately lower level, although some effects may skip levels, as occurs with Conscious Regulation of Emotion, which extends directly from Level 2 to Decision-Making at Level 4. These findings are consistent with the study by Ramos-Galarza et al. (2026) in Chilean, Cuban, and Ecuadorian populations, which also revealed significant interrelationships between behavior regulation and learning verification.

Given the central nature of Conscious Regulation of Behavior, its moderate internal consistency (*α* = 0.65; *ω* = 0.69) should be considered, which is consistent with other studies in Cuban samples (Díaz-Guerra et al., 2025a; Ramos-Galarza et al., 2026) and is comparable to that reported in the original Chilean-Ecuadorian validation (*α* = 0.76; *ω* = 0.74; Ramos-Galarza et al., 2023). Despite these moderate coefficients, several indicators suggest that this dimension contributes stably to the structural model. The factor loadings of its items are significant in the configural and metric invariance analyses, indicating that the factor is well defined by its indicators. Furthermore, in the final structural model, Conscious Regulation of Behavior presents the highest coefficient of determination of all endogenous variables, demonstrating that its predictors explain a substantial proportion of its variance. This dimension also acts as the most connected node of the system, in addition to being a critical mediator in the transmission of effects from metacognition to lower-level executive functions. These pieces of evidence suggest that, despite the moderate reliability estimates of the dimension, its inclusion in the model is justified by its theoretical and empirical role in the hierarchical organization of executive functions.

It is important to note that the dimensions of the UEF-1 should not be interpreted as direct one-to-one equivalents of the core components typically identified in laboratory models, such as inhibition, updating of working memory, and cognitive flexibility or attentional shifting. Rather, they represent ecologically embedded manifestations of executive control as expressed in the academic life of university students. Within this framework, inhibition is most closely represented by Conscious Regulation of Behavior, which involves suppressing impulsive responses, anticipating consequences, and adjusting behavior to situational demands. It is also indirectly supported by Conscious Regulation of Emotions, insofar as emotional stability reduces impulsive reactivity, and by the Supervisory Attentional System, which contributes to the control of internal and external distractors. Processes related to working memory are primarily reflected in the Supervisory Attentional System and Behavioral Verification for Learning. These dimensions involve maintaining task goals, sustaining attention, monitoring academic progress, and keeping relevant information active while completing or reviewing academic tasks. Cognitive flexibility does not appear as an independent factor in the present model; instead, it is presented as a process distributed across several dimensions, particularly in Decision-Making, Management of Elements to Solve Tasks, Behavioral Verification for Learning, and Conscious Monitoring of Responsibilities. These functions require students to evaluate alternatives, reorganize strategies, adjust resources, and modify their behavior according to changing academic demands.

In this sense, the proposed model is consistent with the unity-and-diversity framework of executive functions (Friedman and Miyake, 2017; Miyake et al., 2000). The significant correlations among all executive dimensions and the hierarchical organization of the model support the idea of unity, suggesting the presence of a shared regulatory architecture underlying academic executive functioning. At the same time, the differentiation into four levels and seven specific functions supports the idea of diversity, indicating that executive control is expressed through partially distinct but interdependent processes. However, the present model differs from the framework of Friedman and Miyake (2017) in two main aspects. First, whereas that model was originally derived from laboratory tasks designed to isolate latent executive components, the present model is based on ecologically valid self-report indicators of executive functioning in real academic contexts. Second, instead of organizing executive functions as parallel latent components, the proposed model describes a hierarchical and functionally sequential architecture. Therefore, the present findings should be understood as complementary to traditional componential models; they do not replace inhibition, working memory, or cognitive flexibility as core executive constructs, but rather show how these processes may be expressed, combined, and organized in the daily academic functioning of university students.

### 4.1 Practical implications for intervention and university support

The four-level hierarchical architecture proposed by the model offers an empirically grounded framework for designing interventions aimed at strengthening executive functions in university students. The sequential organization of the system suggests that interventions could adopt a stepped approach: begin by strengthening higher-level metacognitive processes (Conscious Monitoring of Responsibilities and Behavioral Verification for Learning) through training programs in self-regulation, goal setting, and systematic learning verification habits; continue by addressing emotional and behavioral regulation (Conscious Regulation of Emotions and Conscious Regulation of Behavior), where the former could precede and facilitate the latter; and finally, direct efforts toward sustained attention and operational outputs (Decision-Making and Management of Elements to Solve Tasks). Given that Conscious Regulation of Behavior emerged as the central node of the system (*R*^2^ = 0.724), interventions that succeed in strengthening this function could have cascading effects on the rest of the executive system.

Additionally, the decision trees revealed that predictive relationships were especially strong for values below the mean, suggesting that the model could be useful for early detection of students at risk of executive difficulties. Brief screening instruments based on higher-level executive functions could allow university support services (student welfare, psychopedagogical counseling, academic mentoring programs) to identify students who could benefit from preventive interventions. It is important to emphasize that these implications are proposals derived from the model, not definitive recommendations, and require validation through experimental and intervention studies that evaluate the effectiveness of these strategies and determine whether strengthening higher-level processes produces, as the model suggests, improvements in the lower levels of the executive system.

## 5 Conclusion

The present study constructed and empirically validated a unified explanatory model of executive functions in Cuban university students, specifically as perceived and self-reported in their academic contexts, an approach that captures the subjective experience of executive control and its organization in everyday university life. Using a sequential methodology that integrated decision trees (CHAID) and structural equation modeling (SEM), the results showed that the executive functions assessed are organized into a hierarchical architecture composed of four interdependent levels: Metacognitive Evaluation and Supervision (Level 1), Emotional and Behavioral Regulation (Level 2), Operational Attentional System (Level 3), and Goal-Directed Action and Task Resolution (Level 4). The model achieved satisfactory fit indices (CFI = 0.915, RMSEA = 0.048, SRMR = 0.045), confirming its structural validity.

The model reveals that the executive system shows a pattern of relationships predominantly from metacognition toward action. At Level 1, Conscious Monitoring of Responsibilities and Behavioral Verification for Learning operate as correlated exogenous variables that, in the proposed model, are associated with the initial metacognitive signal. At Level 2, Conscious Regulation of Emotion is structurally positioned prior to Conscious Regulation of Behavior, the latter being the most highly connected and critical node in the system, as it shows convergent associations with three sources and projects effects toward all subsequent levels. Level 3 is configured as an integrative node that receives inputs from four sources, with Conscious Monitoring of Responsibilities emerging as its strongest predictor. Finally, Level 4 have two differentiated operational outputs: Decision-Making, which integrates behavioral, emotional, and attentional influences, and Management of Elements to Solve Tasks, which shows stronger associations with behavioral control and the habit of verification.

The findings of the model have relevant theoretical and methodological implications for the study of executive functions in educational contexts. Theoretically, the model proposes a hierarchical and sequential organization of executive functions as perceived by students in their everyday academic environment, moving beyond previous models focused on bivariate relationships or a limited number of components, and offering an ecologically grounded perspective that captures executive functioning in real-world learning situations. This four-level architecture offers a framework for understanding how higher-order metacognitive processes are related to, and potentially modulate, more basic executive functions in ecologically valid situations, distancing itself from laboratory-based experimental approaches that isolate tasks and contexts. Methodologically, the study demonstrates the value of combining decision trees and structural equation modeling to explore complex relationships among psychological variables, establishing a precedent for future research aimed at constructing explanatory models in educational neuropsychology. Likewise, validation of the model in a large and representative sample of Cuban university students provides evidence regarding the applicability of the UEF-1 in Latin American contexts with specific sociocultural characteristics, which may encourage replication of the model in other Spanish-speaking populations.

## 6 Limitations and future directions

Our results should be interpreted in light of certain limitations that also open avenues for future research. The cross-sectional design prevents the establishment of unequivocal causal relationships among executive functions, a constraint that future longitudinal studies could help overcome. Such studies would be particularly valuable for examining the temporal precedence of the proposed levels and examining how the executive architecture evolves throughout the university trajectory.

The Conscious Regulation of Behavior dimension presented moderate internal consistency coefficients (*α* = 0.65; *ω* = 0.69) in the present study, consistent with previous research in Cuban samples. Although its retention as a factor is justified in the Discussion of the results, we acknowledge that future research should revisit the wording of the items composing this dimension to improve their comprehension and internal consistency in Cuban populations.

Measurement invariance analyses by sex revealed that, although the factor structure (configural invariance) and factor loadings (metric invariance) are maintained across men and women, scalar invariance was not achieved. This implies that the intercepts of some items differ systematically between groups, preventing direct comparisons of latent means across sexes. While this finding does not affect the structural validity of the proposed four-level hierarchical model, nor the predictive relationships among executive functions, which proved to be invariant, future research should explore the sources of this measurement bias. Potential contributing factors include differential interpretation of certain items, response patterns associated with gender norms, or differences in social desirability when reporting emotional or self-regulatory behaviors.

The final model incorporated 14 correlations between item residuals based on modification indices to improve model fit. Although all modifications were theoretically justified and grouped into coherent categories, we recognize that a high number of post-hoc modifications may increase the risk of overfitting and capitalization on chance. To mitigate this concern, a bootstrapping analysis with 2000 replications and a 90% confidence level was implemented, which confirmed the stability of all structural coefficients, none of which presented confidence intervals that included zero. This finding supports the robustness of the model and suggests that the modifications made do not respond to idiosyncratic characteristics of the sample. Nevertheless, we emphasize the need for replication of the model in independent samples from different university contexts and countries to confirm the generalizability of the proposed hierarchical architecture and definitively rule out the possibility of overfitting.

The exclusive use of self-report to assess executive functions constitutes a limitation of our study. We acknowledge that self-reports capture the subjective perception of executive functioning, which may not fully coincide with objective performance on laboratory tasks and may be affected by response biases, such as social desirability, acquiescence effect, or the tendency to respond consistently. However, it is also recognized that self-reports offer unique advantages for the study of executive functions in ecological contexts, as they capture executive functioning as it manifests in real-life situations and reflect the individual’s perceived effectiveness, an aspect that laboratory tasks cannot capture.

Although the present model provides an ecologically valid description of executive functioning in university students, future studies should consider integrating self-report measures with performance-based tasks and observer reports to obtain a more comprehensive picture of executive functioning. As Barkley (2012) suggests, the extended phenotype model implies that different levels of executive functioning require different assessment methods. A multimethod approach would allow researchers to examine the convergence and divergence across levels, identify profiles of strength and weakness, and determine which level of assessment is most predictive of real-world outcomes such as academic performance and daily functioning (Toplak et al., 2013). For future research, we recommend complementing self-report with performance measures (such as computerized tasks of inhibition, cognitive flexibility, and working memory) and with reports from external observers (such as professors or peers), which would allow triangulation of information and reduction of biases associated with each method. The inclusion of multimethod measures would enable evaluation of the convergent validity of the proposed hierarchical model and determine whether the four-level structure holds when executive functions are assessed through different approaches. Furthermore, longitudinal designs combining ecological momentary assessment with periodic performance-based testing could capture the dynamic interplay between executive capacities and contextual demands over time, providing a richer understanding of how executive functioning unfolds in naturalistic academic contexts.

Finally, the correlational nature of the structural equation analyses, although methodologically robust, does not definitively prove the directionality proposed by the model. For this reason, experimental and intervention studies are needed to test the practical applicability of the proposed hierarchical architecture through the design and implementation of training programs aimed at strengthening the processes identified as critical nodes of the system. Such studies should evaluate whether these interventions produce transfer effects toward other levels of executive organization. Additionally, replication of the model in other Spanish-speaking university populations would make it possible to assess the cross-cultural invariance of the findings and their potential generalizability to diverse educational contexts.

## Data Availability

The datasets presented in this study can be found in online repositories. The names of the repository/repositories and accession number(s) can be found in the article/supplementary material.
